# Supplementary material for: The Application of Quantitative Metabolomics for the Taxonomic Differentiation of Birds
Source: Biology (Basel). 2022 Jul 21;11(7):1089. doi: 10.3390/biology11071089 (PMC9312993; doi:10.3390/biology11071089)
Supplement: Supplementary file 1 [file biology-11-01089-s001.zip › Supplementary information.pdf]

Supplementary information

for

Application of quantitative metabolomics for taxonomic differentiation of birds

by

Ekaterina A. Zelentsova[1], Lyudmila V. Yanshole[1], Yuri P. Tsentalovich[1], Kirill A. Sharshov[2], and Vadim. V. Yanshole[1,*]

[1] *Laboratory of Proteomics and Metabolomics, International Tomography Center SB RAS, Institutskaya 3a, Novosibirsk, 630090, Russia*

[2] *Laboratory of Molecular Epidemiology and Biodiversity of Viruses, Federal Research Center of Fundamental and Translational Medicine, Timakova str. 2, Novosibirsk, 630117, Russia*

[*] *Corresponding author. E-mail address:* vadim.yanshole@tomo.nsc.ru

Contents:

Supplementary Figure S1. Representative ^{1}H NMR spectrum of bird lens metabolome.

Supplementary Figure S2. PCA scores plots for non-scaled (left panel) and Pareto-scaled (right panel) data.

Supplementary Figure S3. HCA clustering results.

Supplementary Figure S4. HCA dendrograms obtained for non-scaled (left panel, NSS) or Pareto-scaled (right panel, PSS) data.

Supplementary Figure S5. Clustering result shown as heatmap.

Supplementary Table S2. Average concentrations of metabolites in 14 bird species in nmol/g, color-coded.

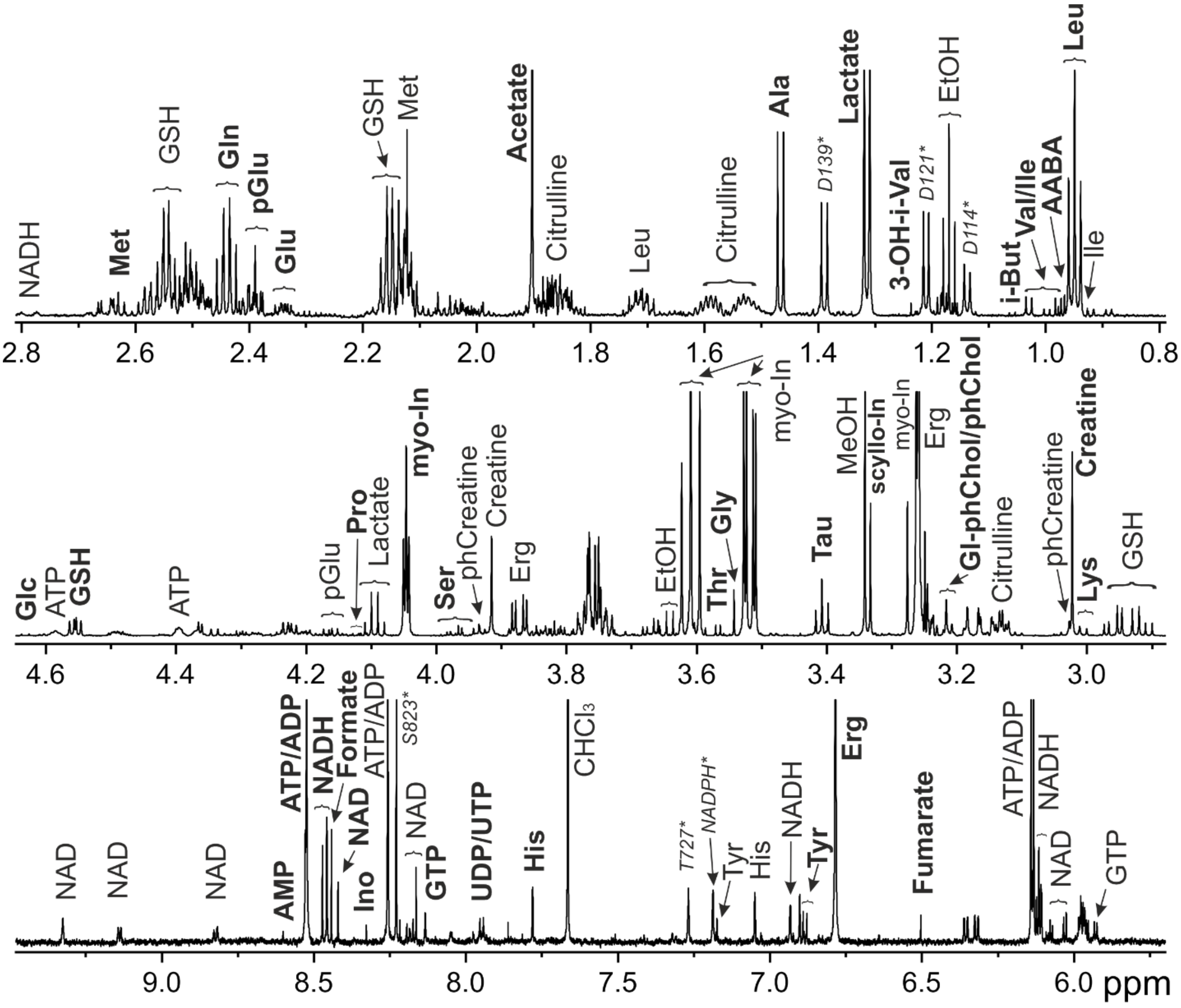

Supplementary Figure S1. Representative ^{1}H NMR spectrum of bird lens metabolome. Obtained for *P. cristatus* lens. The signals used for metabolite quantification are shown in bold. Abbreviations: 3-OH-i-Val – 3-hydroxyisovalerate, AABA – 2-aminobutyrate, ADP - adenosine diphosphate, AMP - adenosine monophosphate, ATP – adenosine triphosphate, CHCl3 – chloroform, Erg – ergothioneine, EtOH – ethanol, Glc – glucose, Gl-phChol – glycero-3-phosphocholine, GSH – glutathione reduced, GTP - guanosine triphosphate, i-But – isobutyrate, Ino – inosine, MeOH – methanol, myo-In – myo-inositol, NAD - nicotinamide adenine dinucleotide, NADH – nicotinamide adenine dinucleotide reduced, NADPH - nicotinamide adenine dinucleotide phosphate reduced, pGlu – pyroglutamate, phChol – O-phosphocholine, phCreatine – phosphocreatine, scyllo-In – scyllo-inositol, Tau – taurine, UDP – uridine diphosphate, UTP – uridine triphosphate. For amino acids, standard tree letter code is used.

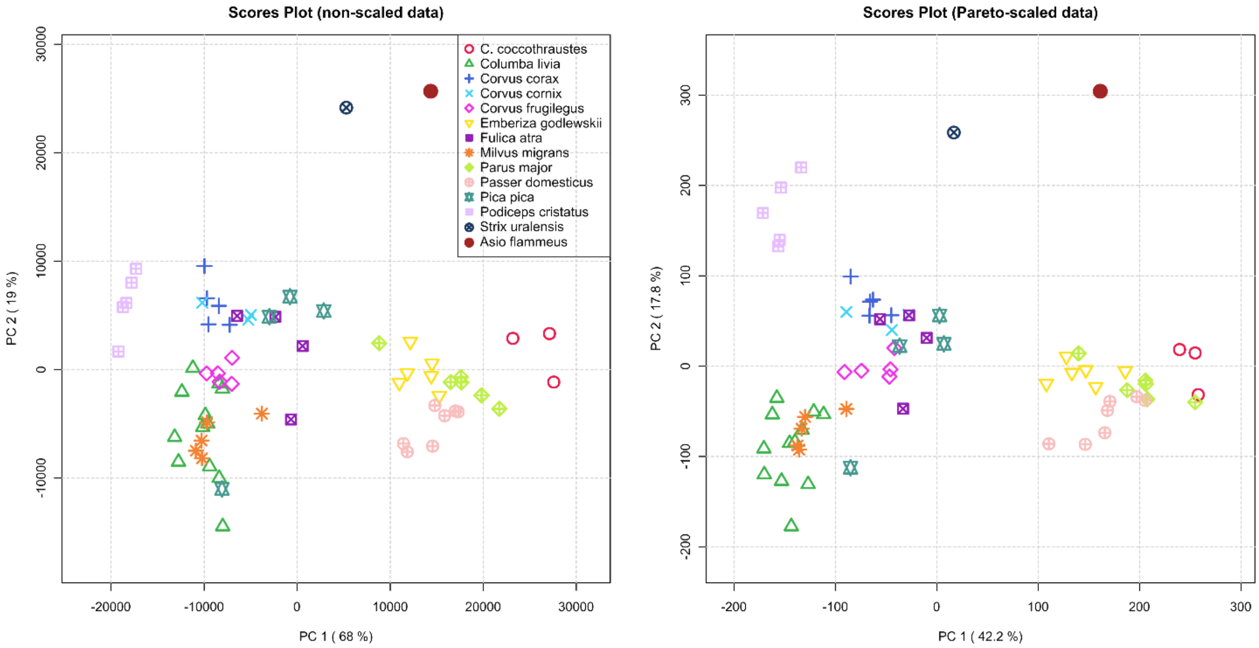

Supplementary Figure S2. PCA scores plots for non-scaled (left panel) and Pareto-scaled (right panel) data.

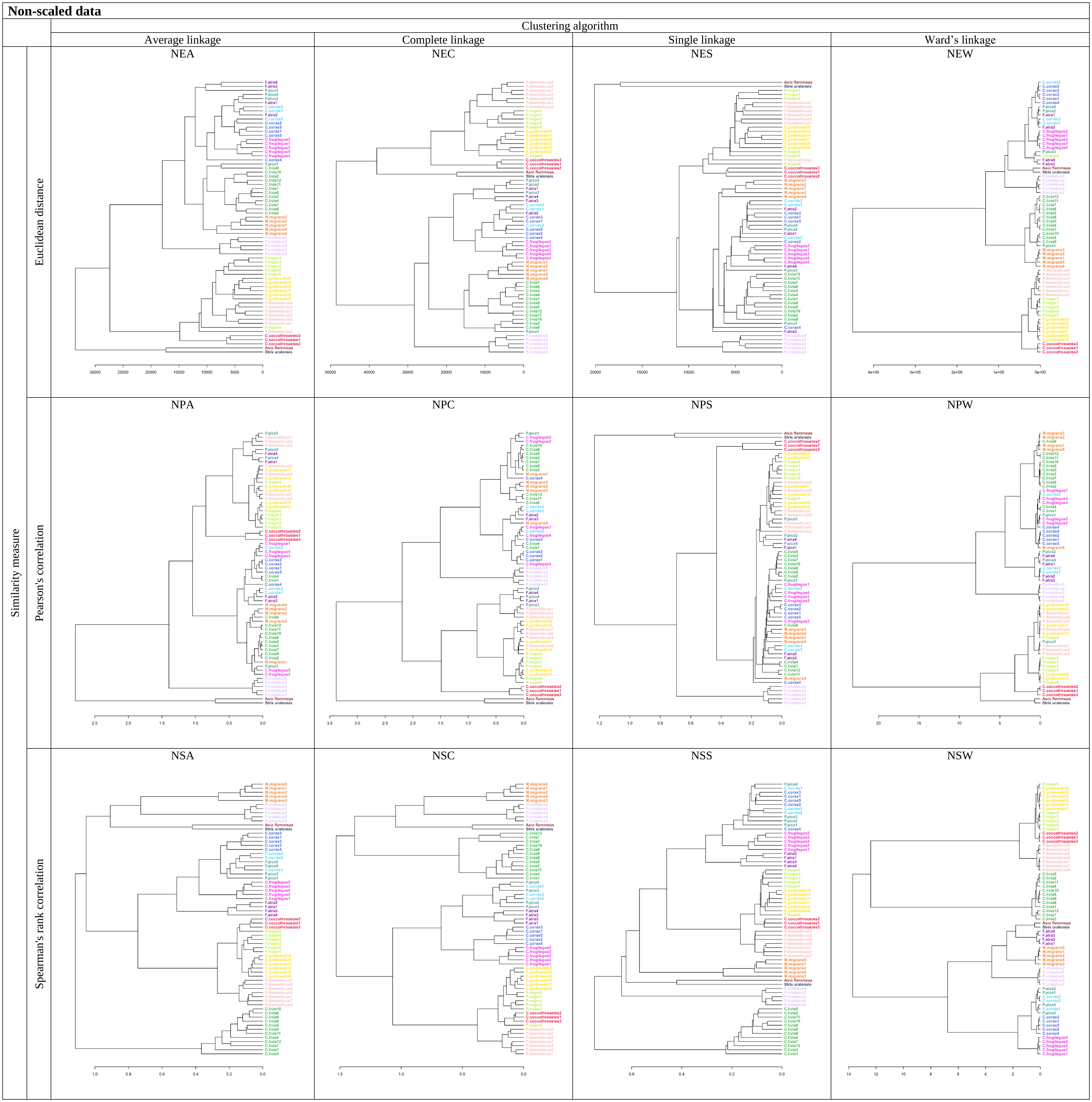
Non-scaled data
Clustering algorithm
Average linkage
Complete linkage
Single linkage
Ward's linkage
Similarity measure
Euclidean distance
Pearson's correlation
Spearman's rank correlation
NEA
NEC
NES
NEW
NPA
NPC
NPS
NPW
NSA
NSC
NSS
NSW

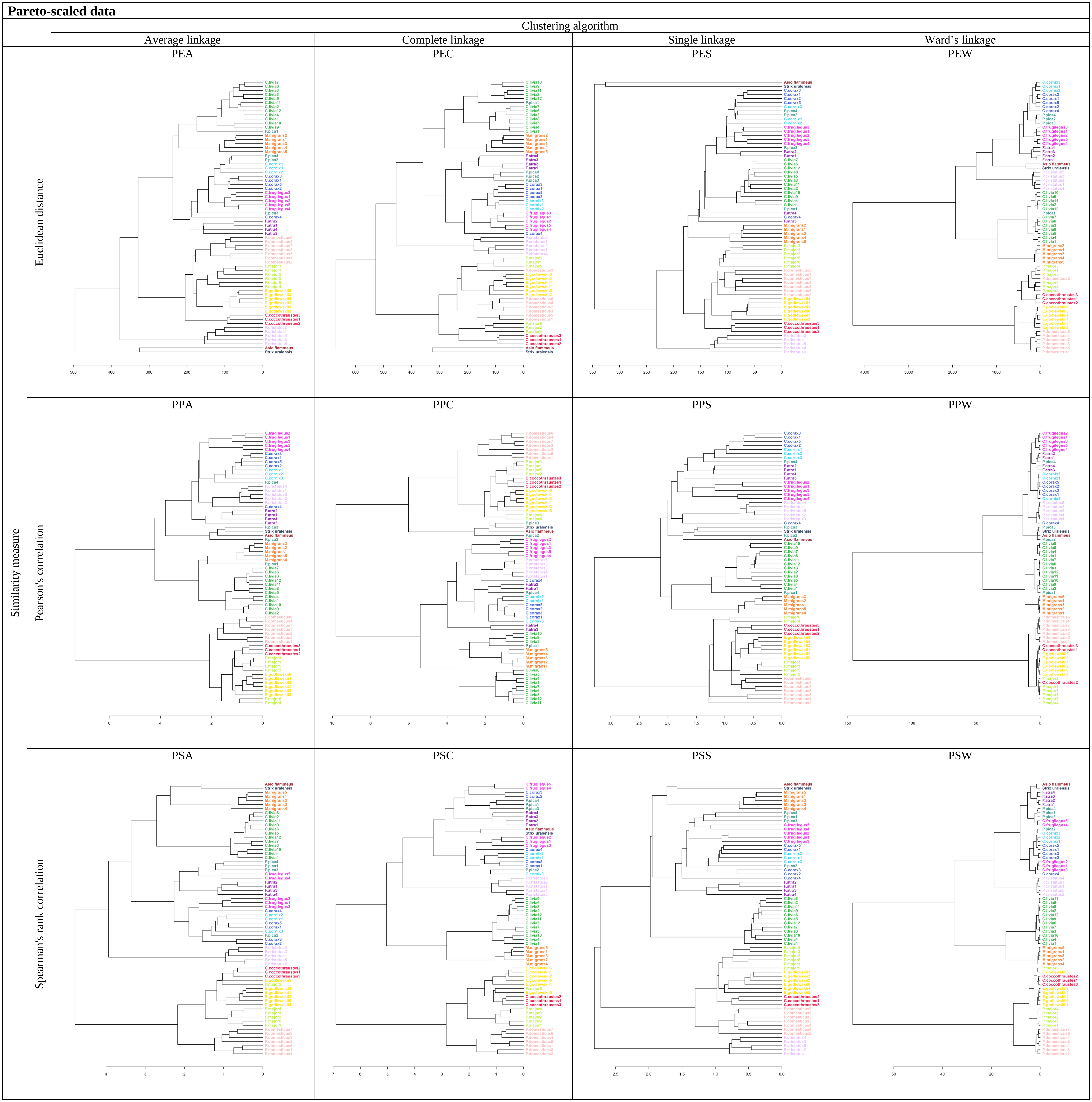
Pareto-scaled data
Clustering algorithm
Average linkage
Complete linkage
Single linkage
Ward's linkage
Similarity measure
Euclidean distance
PEA
PEC
PES
PEW
Pearson's correlation
PPA
PPC
PPS
PPW
Spearman's rank correlation
PSA
PSC
PSS
PSW

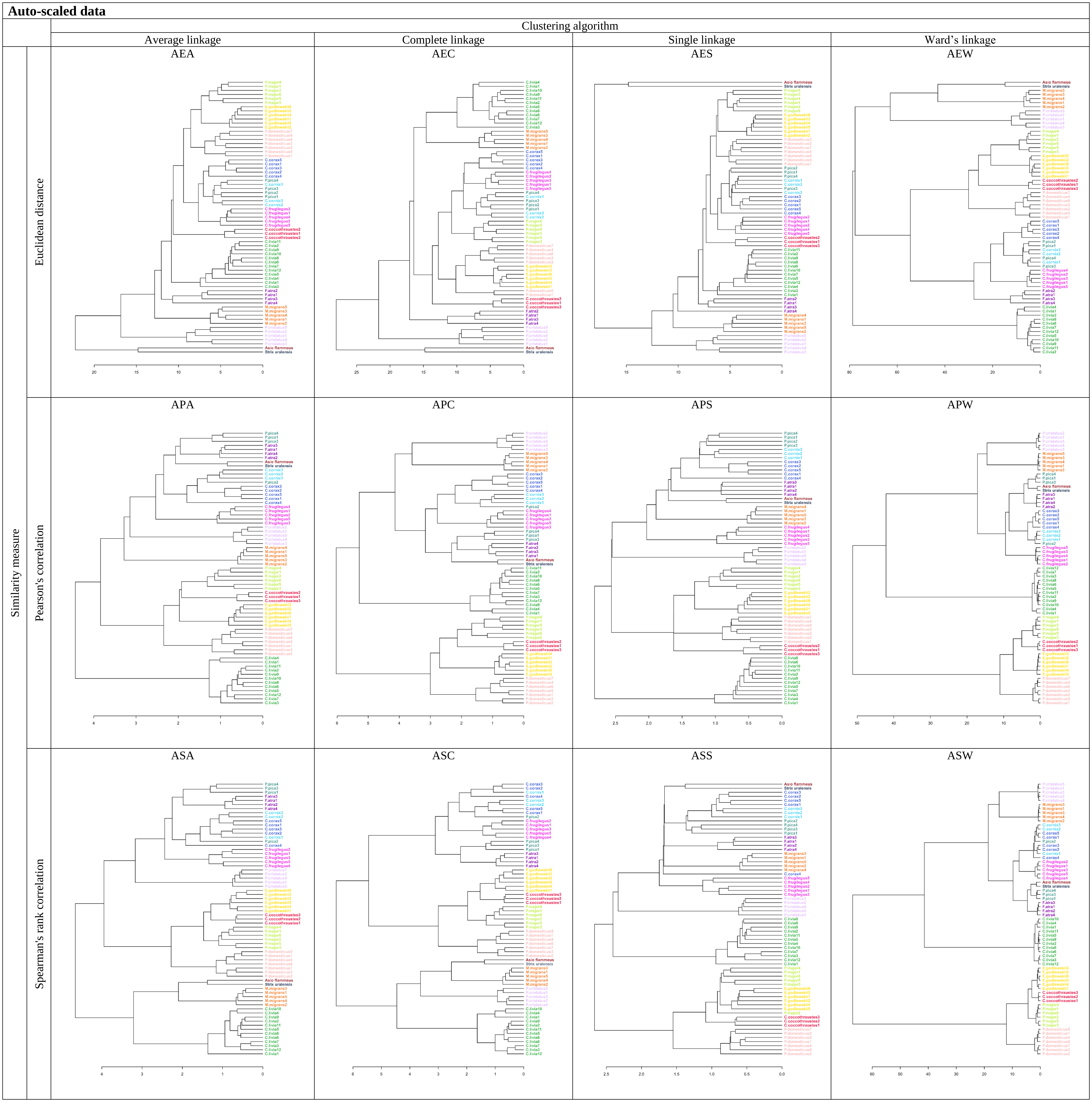

Supplementary Figure S3. HCA clustering results for all 36 possible combinations of data scalings, similarity measures and clustering algorithms, available in MetaboAnalyst web-platform.

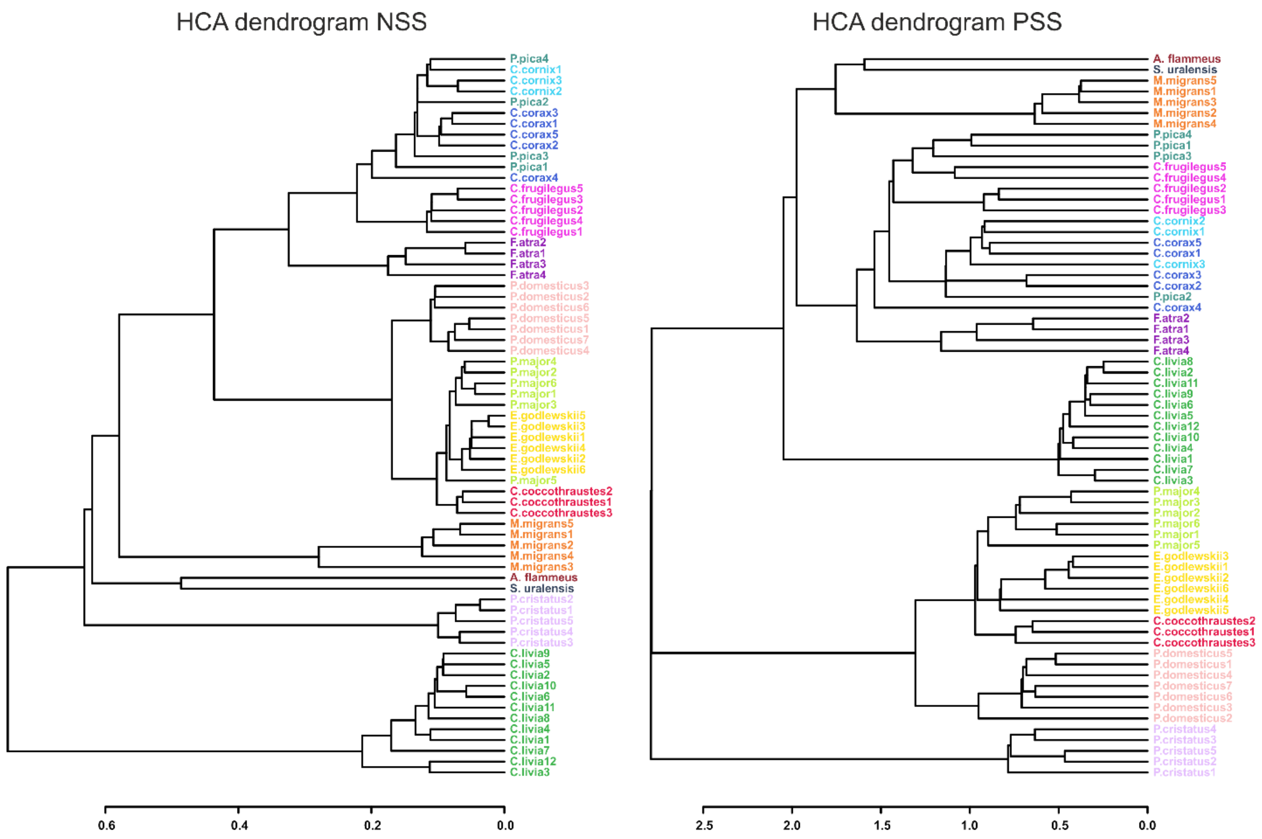

Supplementary Figure S4. HCA dendrograms obtained for non-scaled (left panel, NSS) or Pareto-scaled (right panel, PSS) data. Plotted with the Spearman's rank correlation similarity measure and use of Single linkage clustering algorithm (clustering uses the closest pair of observations).

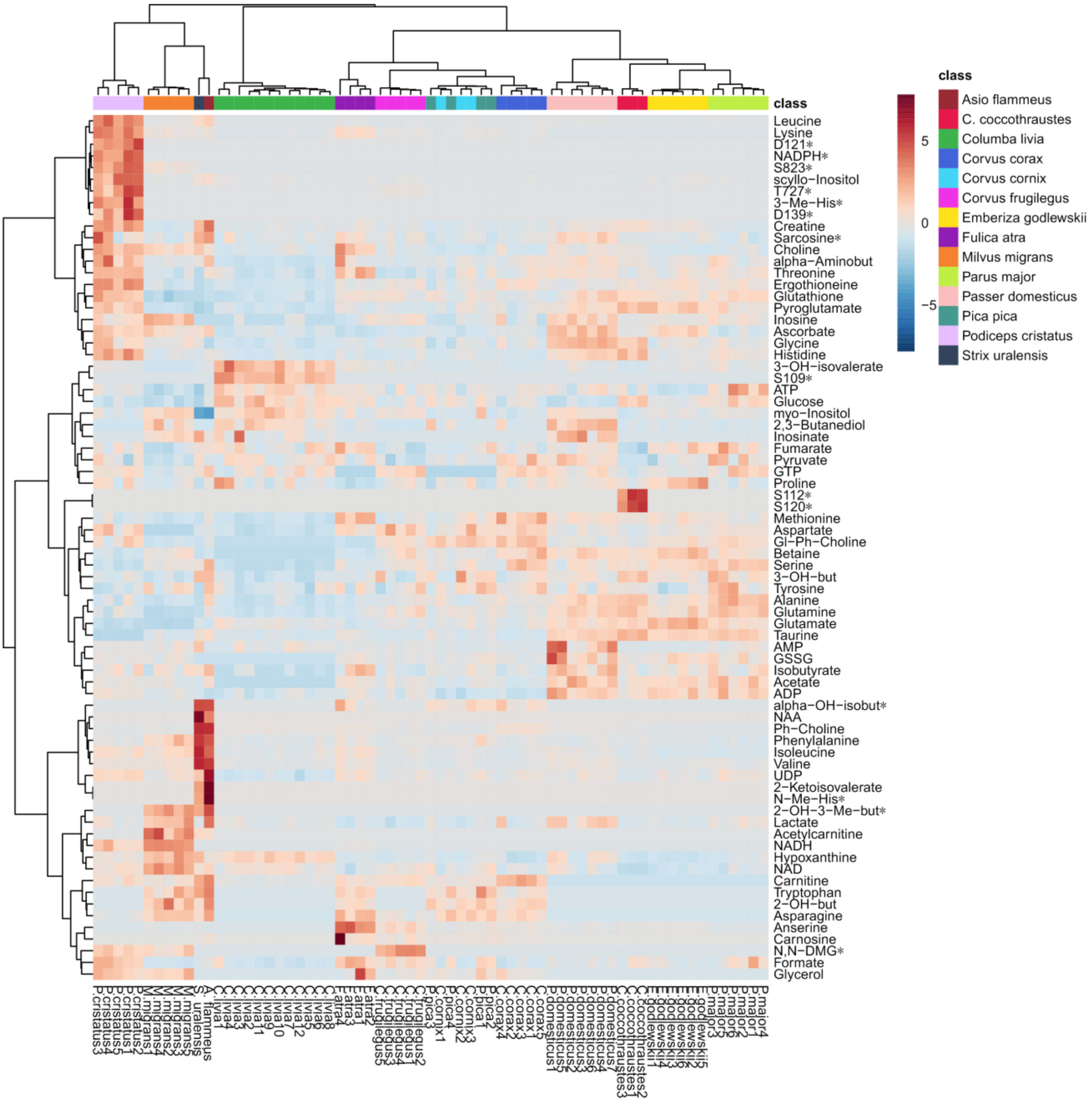

Supplementary Figure S5. Clustering result shown as heatmap. HCA is performed for both – metabolites and samples (Euclidean distance measure, and clustering algorithm using Ward's linkage)

Supplementary Table S2. Sorted average concentrations of metabolites in 14 bird species in nmol/g, color-coded.

Legend for concentration values coloring:

20000	[C] > 10.000 nmol/g	1500	3.000 > [C] > 1.000 nmol/g
5000	10.000 > [C] > 3.000 nmol/g	500	<1000 nmol/g

\ Species<br>Metabolite	Black kite	Eurasian magpie	Northern raven	Eurasian coot	Godlewski's bunting	Great crested grebe	Great tit	Hawfinch	Hooded crow	House sparrow	Rock dove	Rook	Short-eared owl[a]	Ural owl[a]	Average
	M. migrans	*P. pica*	*C. corax*	*F. atra*	*E. godlewskii*	*P. cristatus*	*P. major*	*C. coccothraustes*	*C. cornix*	*P. domesticus*	*C. livia*	*C. frugilegus*	*A. flammeus*	*S. uralensis*	
myo -Inositol	37644	29142	25982	28923	28984	27729	29454	25127	26315	33735	37071	32297	2974	5354	26481
Taurine	14986	20099	12722	20142	34987	3225	37936	47662	15029	36307	14527	13989	32320	25625	23540
Lactate	13884	7065	7580	5467	6415	7719	8070	6130	5297	11539	7060	4882	20575	6728	8458
Glutamine	1741	5755	5385	4431	7814	4609	10687	10618	6362	9249	3229	7374	6111	2403	6126
Acetate	4261	4252	3048	6004	8072	5069	9677	6236	3089	10392	89	4887	7166	3117	5383
Glutathione	1187	3529	2940	3880	3972	5993	4703	4643	3574	4379	2143	3638	1841	1397	3416
Alanine	1709	3027	3219	2106	3641	2371	6012	5664	2637	4672	1705	2916	4919	1160	3268
Ergothioneine	1863	1887	4486	2982	4122	9107	3760	3721	3239	2904	1579	3561	1021	361	3185
Serine	2669	2214	4352	2187	3853	1192	4573	3125	3209	2950	904	2846	5582	2915	3041
ATP[b]	2241	3307	3154	3327	2381	3071	4459	3072	2737	3241	3848	3078	2900	1563	3027
Glutamate	788	1805	1858	1580	3866	1022	2677	2985	2126	2777	2020	1890	1326	1625	2025
Creatine	579	629	751	1010	1095	2752	727	1416	778	1077	811	828	3560	2294	1308
Pyroglutamate	564	801	916	917	1682	1706	1222	1896	1100	1328	651	842	343	174	1010
Glucose	270	1402	819	1389	966	499	1570	1916	949	445	1783	796	958	0	983
Ph-Choline	170	89	548	339	69	114	64	167	96	215	246	203	3918	3946	727
ADP	558	428	381	627	964	718	998	745	298	1090	466	563	287	470	614
Glycine	584	312	533	437	533	896	549	950	472	1119	350	543	153	555	570
Proline	477	324	596	263	1045	683	475	575	692	669	647	563	616	278	565
Betaine	591	378	896	205	1027	226	642	720	465	804	0	647	232	106	496
Methionine	225	429	963	1005	593	347	467	498	547	607	196	377	481	167	493
Threonine	300	304	425	818	439	860	505	374	478	539	261	224	540	123	442
UDP	330	311	296	414	264	487	343	205	266	306	147	275	1551	592	413
Leucine	191	105	142	164	98	2175	134	104	164	165	85	116	954	741	381
Anserine	122	220	285	2449	262	0	73	203	242	150	0	746	28	131	351
GSSG	332	398	284	377	556	352	505	349	256	940	0	280	0	0	331
Ascorbate	391	213	234	123	444	489	188	261	161	546	200	182	482	370	306
Valine	213	68	92	197	103	186	115	95	112	171	107	94	1135	1231	280
3-OH-but	96	304	210	169	253	166	365	444	343	237	91	214	484	484	276
NAD	502	255	148	122	102	248	134	50	134	185	224	213	319	248	206
Histidine	124	97	90	111	144	498	212	431	137	308	85	105	189	98	188
Gl-Ph-Choline	53	302	389	20	234	242	273	140	396	138	0	239	141	41	186
Tyrosine	196	204	200	178	132	125	229	175	208	167	142	167	263	97	177
Formate	75	236	83	325	125	322	218	177	68	129	84	233	153	100	166
GTP	118	0	269	0	145	221	259	112	0	211	190	267	316	202	165
NADH	1236	48	17	86	8	563	13	8	28	16	3	0	0	9	145
Aspartate	0	141	210	178	128	215	156	93	219	127	44	243	100	89	139
Lysine	135	67	62	299	0	912	0	0	65	0	12	77	161	88	134
Phenylalanine	184	101	50	74	39	53	24	49	67	44	41	48	509	578	133
scyllo -Inositol	50	34	45	51	70	749	51	39	44	49	55	54	206	171	119
Hypoxanthine	252	94	50	81	51	125	84	53	66	137	185	73	125	243	116
alpha -Aminobut	63	56	90	131	72	224	97	91	84	118	20	53	192	90	99
Asparagine	170	135	118	195	0	37	0	0	148	0	0	54	258	113	88
Isoleucine	82	34	34	73	24	78	22	29	42	46	36	40	242	315	78
D139[c]	0	0	0	0	0	1085	0	0	0	0	0	0	0	0	78
Glycerol	219	120	99	258	0	240	0	0	71	0	0	70	0	0	77
Carnosine	0	60	41	547	0	0	0	0	60	0	0	98	117	27	68
D121[c]	0	0	0	0	0	947	0	0	0	0	0	0	0	0	68
AMP	60	28	11	31	12	51	60	10	10	284	48	39	0	228	62
NAA	0	0	0	0	0	0	0	0	0	0	0	0	274	501	55
S823[c]	102	0	0	0	0	666	0	0	0	0	0	0	0	0	55
Carnitine	43	39	100	51	0	52	0	0	40	0	27	57	144	112	48
3-OH-isovalerate	0	34	22	66	48	51	51	10	24	44	192	47	45	21	47
Tryptophan	71	95	31	65	0	0	0	0	37	0	0	0	161	128	42
Choline	54	11	19	78	13	85	20	32	9	34	18	22	41	69	36
2,3-Butanediol	91	56	31	21	19	0	11	11	10	132	78	0	0	0	33
Inosine	89	17	27	24	34	52	42	29	18	66	8	44	0	0	32
T727[c]	0	0	0	0	0	399	0	0	0	0	0	36	0	0	31
Fumarate	13	23	19	36	29	24	32	10	18	24	25	14	27	26	23
NADPH[c]	0	0	0	0	0	302	0	0	0	0	0	0	0	0	22
N-Me-His[c]	0	0	0	0	0	0	0	0	0	0	0	0	198	80	20
Sarcosine[c]	7	4	8	15	8	22	6	7	8	18	7	10	35	30	13
alpha -OH-isobut[c]	0	11	11	14	0	0	0	0	12	0	0	0	60	61	12
Pyruvate	7	9	16	12	9	11	16	7	11	15	13	7	9	14	11
Inosinate	26	0	0	0	0	0	0	0	0	42	15	0	11	58	11
2-OH-but	28	18	16	14	0	0	0	0	16	0	0	0	40	13	10
N,N-DMG[c]	30	0	0	0	0	39	0	0	0	0	0	72	0	0	10
Isobutyrate	8	7	5	15	9	10	10	7	8	16	2	4	19	11	9
S120[c]	0	0	0	0	0	0	0	126	0	0	0	0	0	0	9
Acetylcarnitine	60	0	0	0	0	0	0	0	0	0	0	0	19	22	7
3-Me-His[c]	0	0	0	0	0	100	0	0	0	0	0	0	0	0	7
S109[c]	0	0	0	0	0	0	0	0	0	0	95	0	0	0	7
S112[c]	0	0	0	0	0	0	0	81	0	0	0	0	0	0	6
2-OH-3-Me-but[c]	18	0	0	0	0	0	0	0	0	0	0	0	31	18	5
2-Ketoisovalerate	0	0	0	0	0	0	0	0	0	0	0	0	21	9	2

[a] Lens from only one individual was analyzed

[b] **abbreviations:** 2-OH-3-Me-but – 2-hydroxy-3-methyl-butyrate; 2-OH-but – 2-hydroxy-butyrate; 3-Me-His – 3-methylhistidine; 3-OH-but – 3-hydroxy-butyrate; 3-OH-isovalerate – 3-hydroxy-isovalerate; ADP – adenosine diphosphate; alpha-Aminobut – alpha-aminobutyrate; alpha-OH-isobut – alpha-hydroxy-isobutyrate; AMP – adenosine monophosphate; ATP – adenosine triphosphate; Gl-Ph-Choline – glycerophosphocholine; GSSG – glutathione oxidized; GTP – guanosine triphosphate; N,N-DMG – N,N-dimethylglycine; NAA – N-acetyl-aspartate; NAD – nicotinamide adenine dinucleotide; NADH – nicotinamide adenine dinucleotide reduced; NADPH – nicotinamide adenine dinucleotide phosphate reduced; N-Me-His – N-methylhistidine; Ph-Choline – phosphocholine; UDP – uridine diphosphate

[c] Low metabolite identification confidence, not confirmed by chemical standards. Concentrations of unknowns were estimated assuming that the signals in the aliphatic part of NMR spectra (S109, S112, S120, D121, and D139) correspond to a single CH_3 group, while the aromatic signals (T727 and S823) correspond to CH group.
